# 3D Printed Mini-Floating-Polypill for Parkinson’s Disease: Combination of Levodopa, Benserazide, and Pramipexole in Various Dosing for Personalized Therapy

**DOI:** 10.3390/pharmaceutics14050931

**Published:** 2022-04-25

**Authors:** Hellen Windolf, Rebecca Chamberlain, Jörg Breitkreutz, Julian Quodbach

**Affiliations:** 1 Institute of Pharmaceutics and Biopharmaceutics, Heinrich Heine University, Universitätsstr. 1, 40225 Düsseldorf, Germany; hellen.windolf@hhu.de (H.W.); rebecca.chamberlain@hhu.de (R.C.); joerg.breitkreutz@hhu.de (J.B.); 2 Department of Pharmaceutics, Utrecht Institute for Pharmaceutical Sciences, Utrecht University, Universiteitsweg 99, 3584 CG Utrecht, The Netherlands

**Keywords:** FDM 3D printing, polypill, Morbus Parkinson, personalized medicine, additive manufacturing, gastro retentive drug delivery

## Abstract

Therapy for Parkinson’s disease is quite challenging. Numerous drugs are available for symptomatic treatment, and levodopa (LD), in combination with a dopa decarboxylase inhibitor (e.g., benserazide (BZ)), has been the drug of choice for years. As the disease progresses, therapy must be supplemented with a dopamine agonist (e.g., pramipexole (PDM)). Side effects increase, as do the required dose and dosing intervals. For these specific requirements of drug therapy, the 3D printing method fused deposition modelling (FDM) was applied in this study for personalized therapy. Hot melt extrusion was utilized to produce two different compositions into filaments: PDM and polyvinyl alcohol for rapid drug release and a fixed combination of LD/BZ (4:1) in an ethylene-vinyl acetate copolymer matrix for prolonged drug release. Since LD is absorbed in the upper gastrointestinal tract, a formulation that floats in gastric fluid was desired to prolong API absorption. Using the FDM 3D printing process, different polypill geometries were printed from both filaments, with variable dosages. Dosage forms with 15–180 mg LD could be printed, showing similar release rates (f_2_ > 50). In addition, a mini drug delivery dosage form was printed that released 75% LD/BZ within 750 min and could be used as a gastric retentive drug delivery system due to the floating properties of the composition. The floating mini-polypill was designed to accommodate patients’ swallowing difficulties and to allow for individualized dosing with an API release over a longer period of time.

## 1. Introduction

Worldwide, about 9% of the world’s population is older than 65 years. Over the next few decades, the UN expects the proportion of older people to continue to rise significantly, so that by 2100 almost 23% of the population will be at least 65. In the EU, the aging process is already more advanced; in 2020, more than 20% of the EU population was 65 years and older [1,2,3]. Due to the increase in susceptibility to disease with age, approximately 50% of Rx-medications are prescribed to patients older than 65 years [4,5,6,7,8]. The average geriatric patient (≥65 years) takes 8.5 tablets per day at different times [3]. This can lead to complications between the different drugs with potential interactions, but also to a decrease in medication adherence, as certain dosing times and intervals are not adhered to or administration is forgotten [9,10]. To promote patient adherence, community pharmacies frequently offer to blister tablets in pouches or place them in medication boxes for daily use [7]. Also, pharmaceutical manufacturers are trying to produce tablets that contain multiple active pharmaceutical ingredients (APIs) in fixed dosages that are often prescribed together [11,12]. For example, several APIs are prescribed for high blood pressure or cardiovascular diseases, and these are now in just one tablet for ingestion (e.g., Vocado^®^ HCT, Berlin-Chemie AG, with olmesartan, amlodipine and hydrochlorothiazide). Another disease that requires the administration of multiple tablets is Parkinson’s disease. So far, the disease can only be treated symptomatically and must be tailored very precisely to the patient, since here effect and side effect go hand in hand, as both too low and too high dopamine levels can lead to symptoms [13,14,15]. Parkinson’s disease is the second most common neurodegenerative disease. On average, patients are diagnosed with Parkinson’s at around 60 years of age. However, the onset is probably preceded by decades of changes in the body. The number of patients worldwide has increased from 2.5 million in 1990 to 6.1 million in 2016. The main cause is the increasing aging of the population. However, the incidence of the disease has also increased by more than 20% within individual age groups during this time [16,17]. Parkinson‘s disease is characterized by progressive degeneration of dopaminergic neurons in the substantia nigra [18,19,20,21,22]. This results in an imbalance in the transmitter system with disinhibition of cholinergic neurons and increased glutamatergic activity (dopamine deficiency and excess of acetylcholine). This results in inhibition of movement. Due to the lack of dopamine, akinesia and bradyphrenia develop, rigor and tremor are consequences of the disinhibited cholinergic system. The disease advances in a progressive manner, showing a stepwise course associated with various motor, behavioral, and psychological disabilities. Therapy begins early with the diagnosis. Suitable APIs and API-classes are: levodopa (LD) (always in combination with dopa decarboxylase inhibitors (DDI, e.g., benserazide, carbidopa)), dopamine agonists (DA, e.g., pramipexole, ropinirole), monoamine oxidase B (MAO-B) inhibitors (selegiline, rasagiline), cathechol-O-methyl transferase (COMT, entacapone, tolcapone) inhibitors, N-methyl-D-aspartate (NMDA) agonists (e.g., amantadine), and anticholinergics (biperidine). For patients <70 years of age (biological age), DA are the drug of choice. In patients >70 years, LD combined with DDI is the preferred therapy [23,24]. As the disease progresses, however, it becomes more difficult to control symptoms by taking tablets alone [21,25,26,27,28]. The effect of the medication then sets in increasingly later and does not last as long: The optimal range of action in which a drug is available in the desired concentration in the body and has the intended effect decreases. Phases with good mobility (ON phases) and with under-mobility (OFF phases) thus become more and more prominent. Non-motor symptoms such as behavioral changes or depression may also become more apparent [24,29,30]. That is why the therapy of Parkinson’s patients is constantly adapted and rarely remains a monotherapy. In the later course, DA and LD are often combined. As patient suffering increases, pharmaceutical manufacturers are trying to develop dosage forms that can alleviate suffering. Thus, there are intestinal pumps (Duodopa^®^, Lecigon^®^, [31,32,33]), transdermal therapeutic systems (TTS, Neupro^®^), orodispersible films and tablets (ODF, ODT [34,35]), tablets, capsules, and floating dosage forms (Madopar^®^ HBS [36,37]) for therapy on the market or in clinical trials. Various research groups are also working on improved therapy [38]. Accordion Pill^®^ is one of the new innovative dosage forms [39]. It contains LD and carbidopa (DDI) in a novel drug delivery system with combined immediate release (IR) and sustained release (SR) kinetics. The design allows gastric retention and thus improved API uptake for Parkinson‘s patients. In another approach, nanoparticles are being investigated as oral and nasal dosage forms, as well as a LD powder inhaler [40,41,42,43,44]. Other research groups test microspheres, liposome nanocapsules, and niosomes loaded with DA for the treatment of Parkinson’s disease. The lipophilic formulation is expected to improve transport through the blood-brain barrier to achieve dose reduction, thereby reducing side effects [45,46,47].

As Fused Deposition Modelling (FDM) 3D printing is currently being investigated for many drugs for personalized medicine [48,49,50,51,52,53,54,55,56,57,58,59], some research groups are also interested in printing individual drug dosage forms for Parkinson’s patients with tailored dosages and release profiles [48,49,60,61,62,63]. The layered structure of the geometries from FDM 3D printing and semi-solid 3D printing allows very precise dosage and adjustment of the dose. This allows the required dose to be administered without triggering side effects, even for APIs with a small therapeutic range [63,64]. FDM 3D printing, also called fused filament fabrication, requires a filament, which is previously produced by hot-melt extrusion (HME) from a mixture of API and polymer as matrix. By simply changing the filament during printing, FDM 3D printing enables the use of multiple APIs and polymer matrices in one tablet during one manufacturing step. This offers the advantage of also being able to combine APIs that are incompatible with each other in a combined formulation, as well as being able to individually adjust the release properties of the APIs due to the polymer matrix and surface area to volume (SA/V) ratio [65,66]. For example, Khaled et al. developed a 3D printed polypill with five different drugs in various compartments and two different release profiles for cardiovascular therapy [67].

In our study, we aimed to develop a 3D printed polypill-dosage form containing three APIs with different release kinetics for the therapy of Parkinson’s disease: pramipexole (PDM), levodopa (LD), and benserazide (BZ). In addition, the dosage form should be adapted to the requirements of Parkinson’s patients and thus be easy to swallow, individually dosed, and have the longest possible gastric residence time (GRT) to saturate the transporters in the upper small intestine section with LD over a long period of time to reduce side effects and ON-OFF fluctuations. Levodopa is a precursor of dopamine and is used in the treatment of movement disorders in Parkinson’s disease and restless legs syndrome. The initial dose is 100 mg LD once or twice daily combined with 25 mg BZ. A dose increase should be made every 3rd– 7th day, until a maximum daily dose of 800 mg LD is reached. LD and BZ are dosed in a 4:1 combination. PDM is a dopamine agonist. The initial dose is 0.26 mg pramipexole per day (corresponds to 0.375 mg PDM), the lowest dose of one tablet is 0.088 mg. The daily dose may be increased by 0.52 mg at weekly intervals, to a maximum dose of 3.15 mg per day (corresponds to 4.5 mg PDM) [23]. For individual dosage and adjusted release rate, the FDM 3D printing process was used. The DA PDM should have a fast release and the combination LD/BZ should display sustained release from the dosage form. Therefore, PDM was processed by HME in a polyvinyl alcohol (PVA)-filament and the combination LD/BZ in an ethylene-vinyl acetate-copolymer (EVA)-filament. The dosage form design should be adjusted for the release rate with respect to the absorption window in the upper jejunum via the SA/V ratio. 

## 2. Materials and Methods

### 2.1. Materials

For formulation development, various sustained release (SR) polymers were first screened using the vacuum compression molding (VCM) method (Table 1). 

After formulation development, the polypill was printed with two different filaments, manufactured by hot-melt extrusion (HME). The composition of the filaments is shown in Table 2. 

LD, BZ and PDM exhibit good water solubility (c_s_ (LD) ≥ 12 mg/mL, c_s_ (BZ) ≥ 10 mg/mL, c_s_ (PDM) ≥ 200 mg/mL [68,69,70]) and thus belong to the biopharmaceutical classification system (BCS) class I. As HME and FDM 3D printing are heat intensive processes, care was also taken to ensure that the process temperatures were below the decomposition temperatures (260–330 °C) [62,71,72,73,74]. Due to the high water solubility of the drug substances, the dissolution is governed solely by the polymer properties and not by their solid-state properties.

### 2.2. Methods

#### 2.2.1. Vacuum Compression Molding

**Figure 1:**
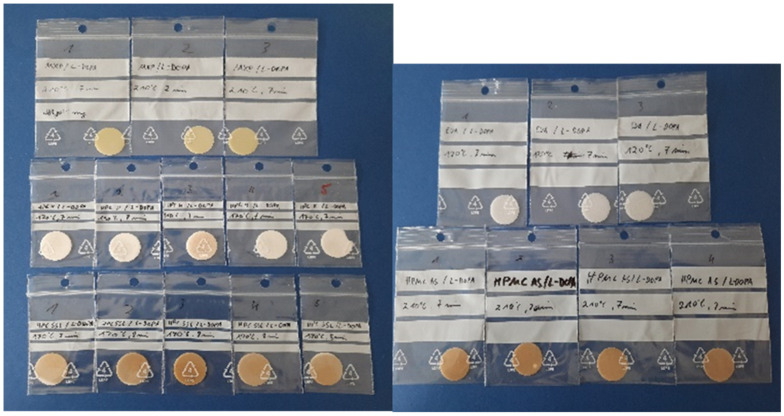
VCM platelets of different SR polymers.

To compare the release profiles of different sustained release (SR) polymers under the same conditions, molten platelets were prepared with vacuum compression molding (VCM, MeltPrep GmbH, Graz, Austria) technology [75]. The resulting platelets had the same surface area (SA) and volume (V), so that the SA/V ratio did not influence the release profile. For this purpose, powder mixtures of different SR polymers with 33% LD each were prepared so that there was 100 mg LD in each VCM-sample (300 mg). The physical mixture of SR polymer and LD was filled into the sample holder, which was connected to a vacuum source. A piston was pressed onto the sample, which was melted on the hot plate until the sample was homogeneously mixed. The process settings used are shown in Table 3. Afterwards, the VCM-platelet was cooled and removed from the holder. The dimensions of the resulting VCM-platelet were 20 mm in diameter and 1.5 mm in height (Figure 1).

#### 2.2.2. Hot-Melt Extrusion for Filament Fabrication

All filaments were prepared by HME with a co-rotating twin-screw extruder (Pharmalab HME 16, Thermo Fisher Scientific, Rockford, IL, USA). A gravimetric feeder (K-SFS-24/6, Coperion K-Tron, Stuttgart, Germany) was used for all experiments. An in-house manufactured die with a diameter of 1.85 mm was used. The desired filament diameter was achieved using a belt haul-off unit of a winder (Model 846700, Brabender, Duisburg, Germany) with a belt speed of 0.8 m/min and the filament was transported through a roller system with four 360°—air flow ring nozzles (Super Air Wipe™, Exair^®^, Cincinnati, OH, USA). With the help of a laser-based diameter measurement module (Laser 2025 T, Sikora, Bremen, Germany), the filament diameter was detected and logged during the process with a readout rate of 1 Hz to ensure the production of filaments with low diameter fluctuations. For extrusions with EVA, the screw speed was set to 20 rpm and powder feed rate was set to 2 g/min. The screw configurations and the temperatures of the heating zones are summarized in Table 4 and also described in previous publications [60,61,76].

#### 2.2.3. 3D Printing Process of the Polypill-Geometries

**Figure 2:**
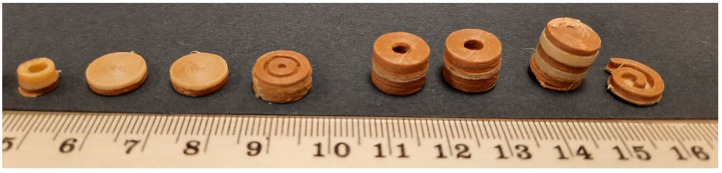
Printed polypills in various designs.

To achieve various dosages and release profiles, the geometries were designed with the computer-aided design (CAD) program Fusion360^®^ (Autodesk, San Rafael, CA, USA) with focus on the volume and surface area to volume (SA/V) ratio. Afterwards, the generated stl-files were transferred to the slicing program PrusaSlicer^®^ (Prusa research, Prague, Czech Republic). The individual parts of the geometries were assigned to the respective filament. The layer height and extrusion width were adjusted to generate the desired height and width of the geometry. The G-code was sent to a Prusa 3D printer (Prusa i3 Mk3, Prusa research, Prague, Czech Republic), which printed the objects defined in the data file (Figure 2). The multi material unit (MMU) from Prusa^®^ was used for printing the polypill. A cleaning tower was printed between filament changes so that the previous filament could be washed out of the nozzle and the following used filament was not contaminated. The best results were obtained with the following temperatures: PDM-PVA filament: 185 °C print temperature and 70 °C bed temperature, LD/BZ-EVA-filament: 220 °C print temperature and 70 °C bed temperature. Cooling during printing was turned off, otherwise the layers would not adhere to each other. The objects were printed one by one. The printing speed was set to 10 mm/s because the geometries had little contact area with the print bed due to their small size and quickly detached, interrupting the printing process.

#### 2.2.4. Dissolution Tests of the Polypills

The dissolution tests for the polypill (*n* = 3) were performed according to European Pharmacopoeia monographs 2.9.3 and 5.17.1 [77,78]. A modified basket apparatus was used for the dissolution apparatus (DT 700, Erweka, Langen, Germany) [61,63]. Adapted baskets were 3D printed with water insoluble polylactide acid filament (PLA, Bavaria-Filaments, Freilassing, Germany) with a mesh size of 3 mm and the same outer dimensions as the regular baskets described in the European Pharmacopoeia. This adjustment was necessary because the 3D printed tablets clogged the small meshes of the original Erweka baskets (0.36–0.44 mm) with swollen polymer, affecting the hydrodynamics around the printed tablet. The use of the modified baskets prevented this blockage. In addition, a 3D printed PLA-plate with a mesh size of 3 mm was clipped into the basket above the floating dosage form so that it could not stick to the stirrer and thus distort the release profiles. As dissolution medium degassed 0.1 N hydrochloric acid (HCl) was used. The volume was 1000 mL, the stirring speed was set to 50 rpm and the temperature was set to 37 ± 0.5 °C. The dissolution tests were performed under sink conditions [63,76]. Samples were drawn using an autosampler (Vision^®^ AutoFill™ + AutoPlus™, Teledyne Hanson Research, Chatsworth, CA, USA). At the set time point, 5 mL were withdrawn from the vessel, 3.5 mL were used to wash the tubes before sampling, and 1.5 mL were transferred directly to a HPLC vial. For polypill design (PP) 1-PP3, the first sample was drawn after 15 min, then after 30 min, and subsequently every 30 min until 180 min. Afterwards, a sample was taken every hour until 360 min, then every 2 h until 600 min. For PP3 additional samples were taken after 600 min every 5 h until 50 h. For the mini tablet designs *MiniTab* and *MiniHC*, the first sample was taken after 10 min, then every 10 min until 60 min, followed by every 30 min to 120 min, then after 1 h to 240 min, and every 2 h to 600 min. Subsequently, samples were taken every 5 h to 1500 min.

#### 2.2.5. HPLC Method: Chromatographic Conditions for Simultaneous Quantification of Levodopa, Benserazide and Pramipexole

The following method is described in more detail in [76]. High performance liquid chromatography (HPLC) analysis was used to separate all three APIs (PDM, LD, BZ). The HPLC (Dionex, Sunnyvale, CA, USA) was equipped with a quaternary pump (P 580 A, Dionex, Sunnyvale, CA, USA) and an autosampler (ASI-100, Dionex, Sunnyvale, CA, USA). For the HPLC method, a C18-column (Eurospher II 100-5, Knauer, Berlin, Germany) with integrated precolumn was used. The eluent consisted of methanol (mobile phase B) and ammonium acetate buffer (0.05 M, pH 4). The flow rate was set to 1 mL/min and the oven temperature for tempering the column to 40 °C. The gradient was as follows: mobile phase B was increased from 1 to 5% (*v*/*v*), within the first min, held at 5% (*v*/*v*) for 4 min, increased from 5 to 10% (*v*/*v*) within 1 min, held at 10% (*v*/*v*) for 4 min, increased again from 10 to 20% (*v*/*v*) within 1 min, held for 4 min at 20% (*v*/*v*), increased again from 20 to 99% (*v*/*v*) within 5 min, held for 2 min at 99% (*v*/*v*) and decreased to 1% (*v*/*v*) within 0.5 min, again until 22.5 min after sample injection. An equilibration time of 3.5 min per run was allowed to pass before the next sample was injected. An injection volume of 200 µL was chosen to analyze the APIs. Detection was achieved by measuring the UV absorption of the sample at 264 nm with the help of the HPLC UV-detector [77].

#### 2.2.6. Density Measurements with Helium Pycnometer

To determine the true density of the filaments and printed tablets, measurements were made using a helium pycnometer (AccuPyk 1330, Model 133/00010/10, Micromeritics, Norcross, GA, USA). The analysis conditions were 10 cycles with a purging filling pressure of 134.55 kPa with Helium. 5 measurements per sample were performed in a 1 cm^3^ chamber.

#### 2.2.7. Comparison of Release Profiles

##### Mean Dissolution Time

The *Mean Dissolution Time (MDT)*, expressed in units of time, was used to compare the curves and to categorize them [61,79,80]. The ***MDT*** was calculated according to Equation (1).

(1) $$MDT=\frac{ABC}{c_{\infty }}=\frac{\sum_{i=0}^{\infty }\left[\left(c_{i+1}-c_{i}\right)\times \frac{\left(t_{i}+t_{i+1}\right)}{2}\right]}{c_{\infty }}$$

The quotient of the ABC (*area between the curves*) and $c_{\infty }$, the initial drug load of the dosage form results in the MDT. Via the trapezoidal equation, ABC is calculated with $c_{i}$ as the concentration of the API released over time t. Values up to 100% API release were used, since the ABC does not change afterwards.

##### Similarity Factor

In addition, the similarity factor was used to compare the release curves. Equation (2) was used to perform the calculation [61,79,81,82].

(2) $$f_{2}=50\times log\left\{{\left[1+\frac{1}{n}\sum_{t=1}^{n}{\left(R_{t}-T_{t}\right)}^{2}\right]}^{-0.5}\times 100\right\}$$

$R_{t}$ represents the API in % at time point t for the reference and $T_{t}$ the API in % at time point t for the test product. The factor n summarizes the considered number of time points. Since the $f_{2}$ value is sensitive to the number of measurement points, the number of the considered values was constantly limited to 12 time points. An $f_{2}$ value of 100 results if the dissolution curve of the test product is completely identical to the reference curve. The measured values may deviate from the reference by a maximum of 10%, resulting in $f_{2}$ values between 50–100. If the achieved $f_{2}$ value is below 50, the dissolution profiles differ strongly, and they are not considered similar.

## 3. Results and Discussion

### 3.1. Polymer Selection for Levodopa

**Figure 3:**
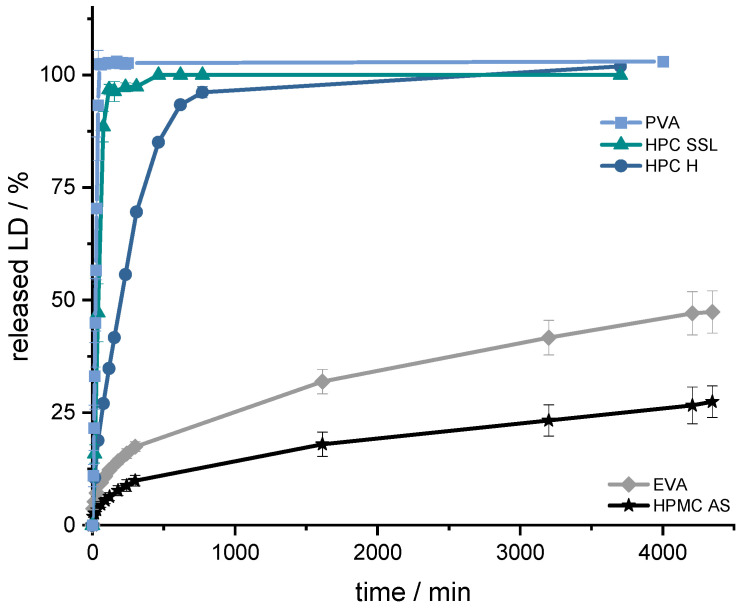
Dissolution profiles of LD from SR-polymer-VCM platelets (33% (*w*/*w*) LD-loading); modified basket apparatus, 1000 mL 0.1 N HCl, 50 rpm, 37.0 ± 0.5 °C. x ± s; *n* = 3.

To increase Parkinson’s patients’ adherence to their therapy, the LD/BZ combination should be released slowly over 12–24 h so that dosing intervals increase, and ON-OFF fluctuations decrease. The PVA-formulation with PDM has already been developed for previous studies [60,61,63]. To find a suitable SR polymer for the LD/BZ combination, VCM-platelets were prepared with a drug loading of 33% (*w*/*w*) LD and established SR polymers: PVA, HPC H, HPC SSL, EVA and HPMC-AS. All VCM-platelets had the same SA/V ratio and could thus be compared based on their dissolution properties (Figure 3).

The aim was to achieve prolonged release with a constant dissolution rate of the API. This target was set regarding the prolonged gastrointestinal passage in Parkinson’s patients [83,84] and the resorption window of levodopa in the small intestinal tract via large neutral amino acid (LNAA) transport carrier [85,86]. To achieve continuous availability of LD/BZ in the body, the dosage form should release a constant amount of LD/BZ and saturate the transporters for as long as possible so the “wearing off” phenomenon at the end of dose interval is decreased [87,88,89]. To ensure a constant release, the tablet should release 75% within 12 h, so that a constant API exposition is realized within the desired time frame. Using the VCM-platelets, it was determined that PVA, HPC H and HPC SSL would not be considered because they released the API too fast (HPC SSL 75% LD in 25 min, PVA: 75% LD in 33 min, and HPC H 75% LD in 133 min) based on their high hydrophilicity, the formation of a hydrocolloid matrix, and swelling, as well as eroding properties of the matrix so that the API can be solubilized faster. The final formulation including BZ should have 50% drug-loading and thus become even more hydrophilic, so that the API release will be faster than the VCM-API release. The API release of HPMC-AS, on the other hand, was too slow (25% LD in 63 h), so the decision was made for the SR polymer EVA (50% API in 75 h). In addition, EVA has a lower density (0.95 g/cm^3^) than water and 0.1 N HCl (gastric fluid), so this property can be exploited for a floating, gastro-retentive drug delivery dosage form [86].

### 3.2. Formulation Development with EVA

**Figure 4:**
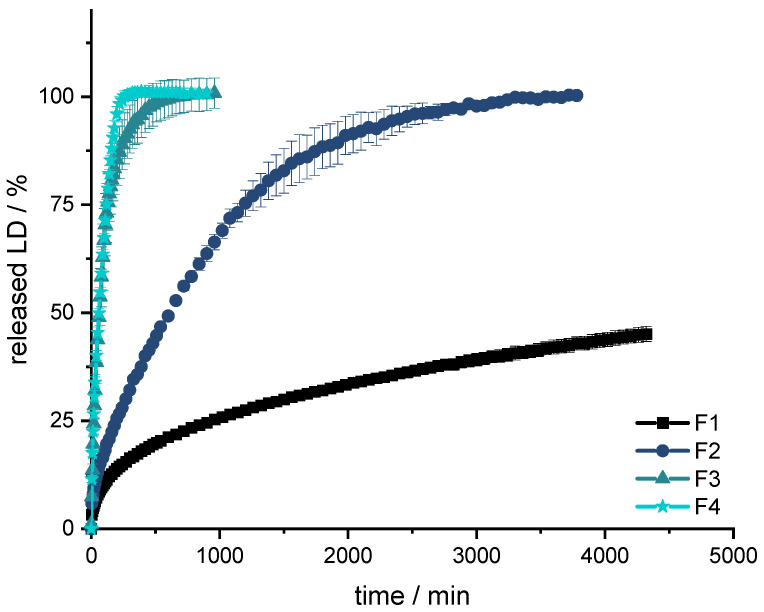
Dissolution of LD from F1, F2, F3 and F4; modified basket apparatus, 1000 mL 0.1 N HCl, 50 rpm, 37.0 ± 0.5 °C. x ± s; *n* = 3.

First, a formulation containing 40% LD and 60% EVA was extruded (F1, Table 5). However, the API release was too slow, even with a high SA/V ratio of 3 mm^−1^ (Figure 4), and the printing process of the filament was difficult, because of the high flexibility of the filament. Therefore, the formulation was changed. PVA was added in equal parts with EVA as hydrophilic polymer (F2). Nevertheless, the flexibility of EVA with a VA content of 28% was too high, so that the printability was poor, the printed objects were not reproducible, and the printing process repeatedly stopped because the filament clogged the nozzle. Drug release was faster than in F1, but still not suitable. Therefore, the EVA polymer with a VA content of 28% was replaced by EVA with a VA content of 18% (F3). The same excipient combination with other quantification was now extruded and printed with EVA (18% VA). Drug release was much faster than with formulation F2, but the dosage form disintegrated within a few minutes, so no gastro-retentive drug delivery form can be developed with this composition. Therefore, the EVA content was increased, PVA was replaced by PVP-VA, and mannitol was added, as the filament otherwise became too brittle (F4). 

All formulations show a burst in the first few minutes. Subsequently, the API is released constantly over time until approximately 80% of LD has been released. Thereafter, the release of LD is slower and results in a plateau. The release profile can be described with Higuchi’s square root-of-time kinetics [90,91,92]. First, the API, which is on the surface of the dosage form, is dissolved. The larger the surface, the more API goes directly into the solution. This results in what is known as a burst. The API is then released from the matrix. In the inert matrix, depending on the diffusion path, the amount of dissolved API remains constant over time. After a certain time, the diffusion paths for the API become longer and longer and less API is released over time until the plateau at 100% is reached. 

The formulations F3 and F4 result in a fast release profile (50% released API in 60 min, 75% released API in 125 min), which may be advantageous when the dosage form is not gastro retentive, and the API must be fully released prior to small intestinal passage. However, since F3 dissolves and does not retain an inert matrix, F4 was used as orientation for the fixed-combination formulation. Formulations F1 and F2 released the API much more slowly (F1: 25% released API in 960 min; F2: 75% released API in 1200 min) and were thus not developed further.

From the obtained results, it could be concluded that the fixed-combination formulation should contain more than 25% EVA for the tablet to remain durable. In addition, PVP-VA was identified as a good pore former and stiffness enhancer for better printability. Since the desired release profile of the fixed combination should still be slower than displayed by F4, the amount of EVA could be increased. 

### 3.3. Formulation Development for Fixed Combination LD/BZ

Based on the previously found formulation with 10% LD, different fixed combinations (FC) were now extruded. The EVA content was set above 30% to produce an inert, non-disintegrating matrix (Table 6). The API proportions were fixed, as they are dosed in a 4:1 ratio (LD:BZ). The maximum dose is 200 mg LD per tablet, which is equivalent to a 500 mg tablet at 40% content, which should be swallowable by patients and designable so that the dimensions of the dosage form are similar to those of tablets on the market. The PVP-VA content varied from 5–20%. The filaments with 20% PVP-VA (FC4) were too brittle and broke directly during cooling after HME, so that they could not be used for printing. The density measurements also reflect the EVA content. The higher the content of EVA in the filament, the lower the density.

**Figure 5:**
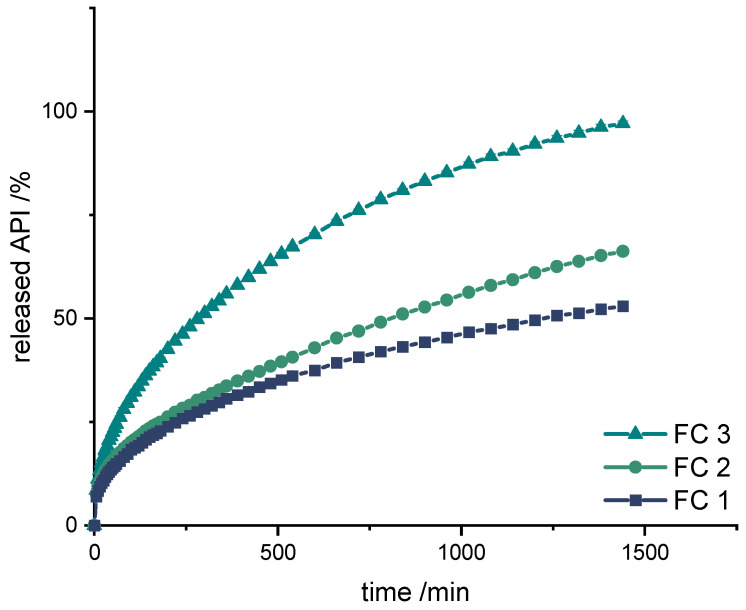
Dissolution of LD/BZ from FC1, FC2, and FC3; modified basket apparatus, 1000 mL 0.1 N HCl, 50 rpm, 37.0 ± 0.5 °C. x ± s; *n* = 3.

After extrusion, parts of the filaments were used for dissolution tests to assess which formulation was most likely to reproduce the desired release profile (SA/V: 2.3 mm^−1^). Parallel quantification of LD and BZ is challenging, and the development of a suitable analytical method to quantify the APIs simultaneous in the presence of PDM is described in another publication [76]. As other publications have already shown, the release profiles of BZ and LD are comparable [93,94,95,96,97]. To simplify the analyses in the present study, the release profile of LD is also used as a surrogate for BZ release (Figure 5).

Also with these formulations, the release starts with a burst effect. Subsequently, the release of the APIs is more uniform. The diffusion paths within the filament strand are very short (Ø 1.75 mm), so that the decrease in the release rate towards the end is small. 

The formulations FC 1+2 release the APIs too slow (FC 1: 50% API in 1260 min, FC 2: 50% API in 780 min), whereas FC 3 shows the fastest release course and displays the desired course (50% API in 290 min, 75% API in 720 min, 100% API in 1440 min).

### 3.4. Design and Dissolution of Polypill Tablet Variations

With the final LD-EVA filament formulation (FC3), and the beforehand developed PDM-PVA filament, different geometries with various PDM and LD/BZ contents were printed and released. The selected doses were adjusted to the dosages in available market preparations. 

**Figure 6:**
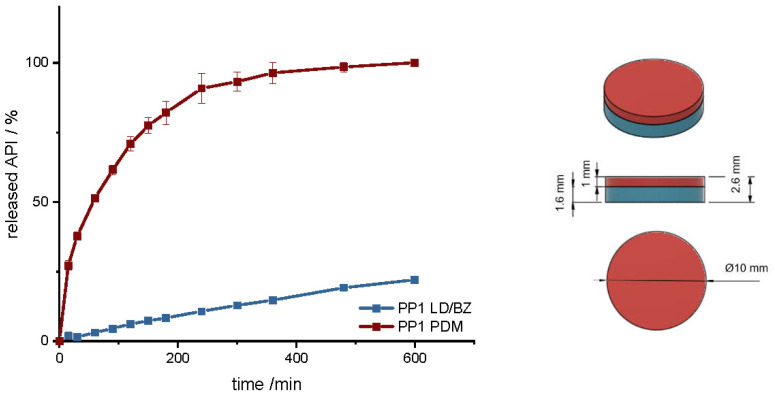
(**Left**): release profile of PP1; modified basket apparatus, 1000 mL 0.1 N HCl, 50 rpm, 37.0 ± 0.5 °C, x ± s; *n* = 3. (**Right**): Image of PP1: red: PDM-PVA, blue: LD/BZ-EVA.

First, a simple polypill design was chosen (PP1) to observe the release behavior of the printed formulation (Figure 6 left). A cylinder with a diameter of 10 mm was selected as geometry, which should therefore also be easy to swallow (Figure 6 right). A LD/BZ dose of 50/12.5 mg was targeted, which corresponds to the lowest dose of tablets available on the market, as well as a PDM dose of 3.5 mg (Table 7). The release rate was calculated for the linear section of the profiles, after the burst until the end of the measurement (LD/BZ), or until the plateau was reached (PDM).

**Figure 7:**
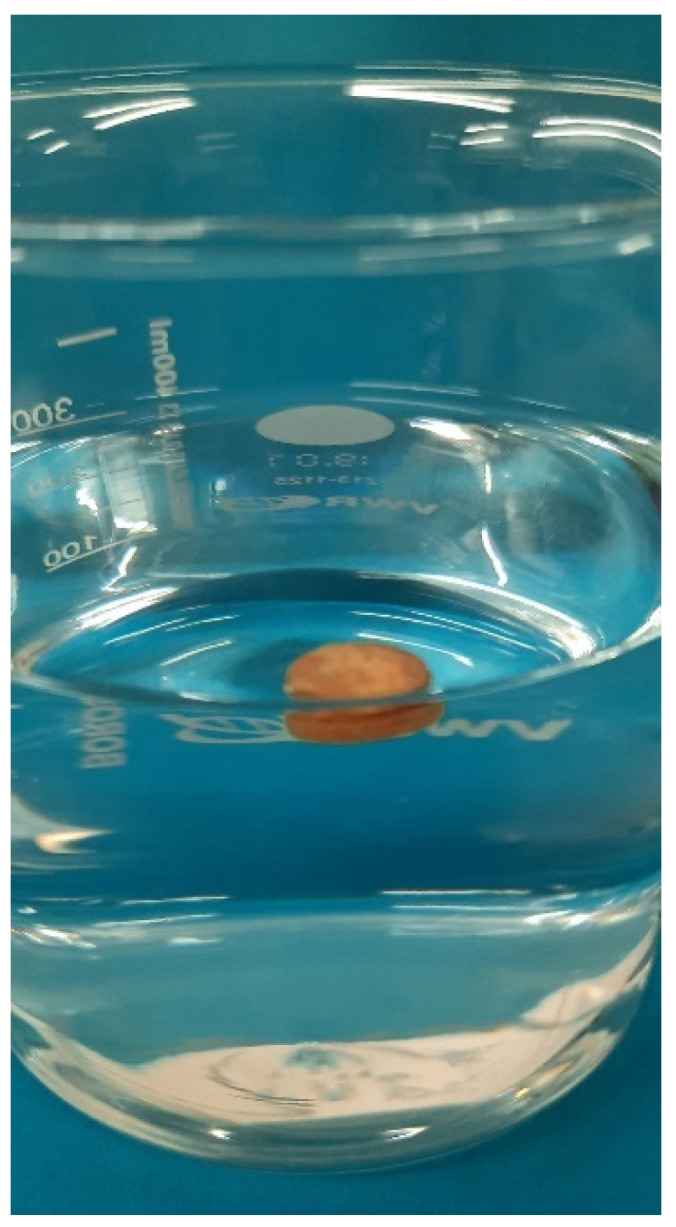
Floating properties of PP1 in 300 mL 0.1 N HCl, 37 ± 0.5 °C.

The geometry has a total SA/V ratio of 1.17 mm^−1^. As the PDM-PVA formulation dissolves over time, the SA of the insoluble LD/BZ formulation increases to 1.65 mm^−1^. The release of PDM can be described by the Peppas Sahlin equation [61]. The formulation releases the API by diffusion and erosion due to the formation of a hydrocolloid matrix [98]. Due to the layered structure of the FDM printed geometry, the medium can easily penetrate the cylinder and release the API from the individual strands. The formulation begins to swell and dissolve over time. The API can release through the layers and dissolve directly due to its good solubility. After 140 min, 75% PDM was released, and thus the dissolution profile can be categorized as prolonged release. The combination of LD/BZ is released very slowly from the SR polymer. The matrix is inert, and the APIs can only enter solution by diffusion. After 600 min, just 22% LD/BZ is released. The density of the entire PP1 is 1.18 g/cm^3^. Due to the low EVA density and most probably included pores, the buoyancy of the polypill is maintained (Figure 7). While the PDM-PVA layer (density 1.3 g/cm^3^) dissolves over time, the remaining EVA-based part retains the floating property.

**Figure 8:**
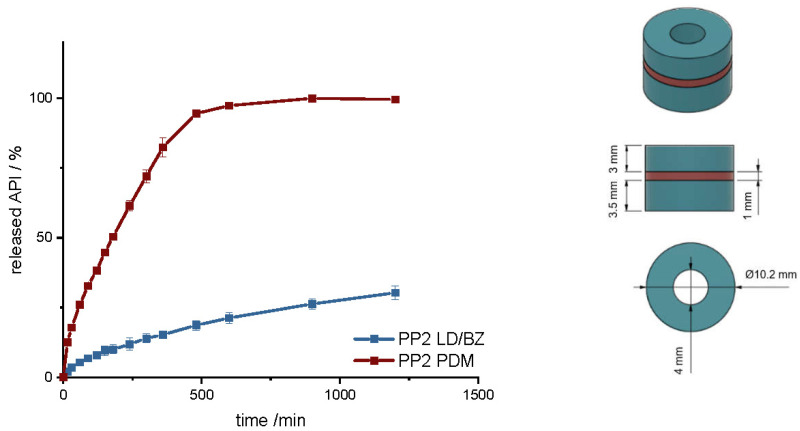
(**Left**): release profile of PP2; modified basket apparatus, 1000 mL 0.1 N HCl, 50 rpm, 37.0 ± 0.5 °C, x ± s; *n* = 3. (**Right**): Image of PP2: red: PDM-PVA, blue: LD/BZ-EVA.

To increase the dose, a hollow cylinder-geometry (PP2) was designed that is built up in three layers, with a total SA/V ratio of 0.9 mm^−1^ (Table 8). The SA/V ratio was kept similar to PP1 to see if it is possible to increase the dose without strongly affecting the overall release. This is of particular importance for personalized therapy [63]. The PDM filament was printed between two LD/BZ-EVA hollow cylinder layers, so that these two hollow cylinders can detach from each other after a while due to the solubility of PDM-PVA-compartment and further increase the SA of the geometry during release to a SA/V ratio of 1.3 mm^−1^ (Figure 8).

**Figure 9:**
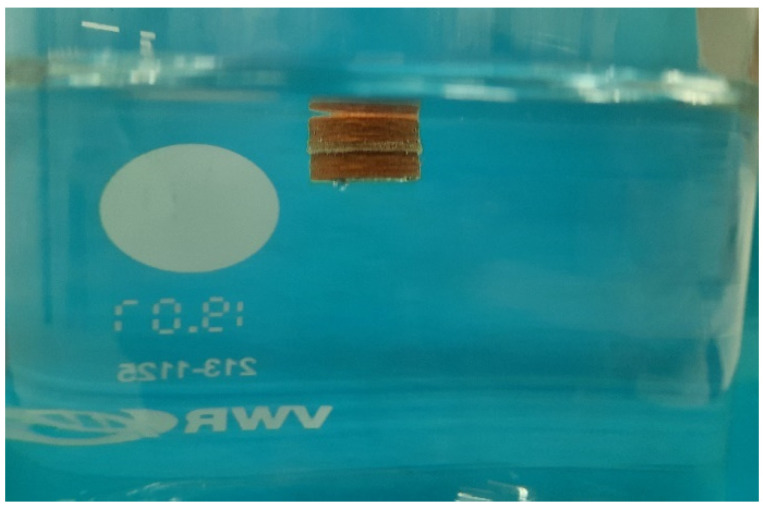
Floating properties of PP2 in 300 mL 0.1 N HCl, 37 ± 0.5 °C.

Compared to PP1, PDM is released more slowly here. This is due to the enclosed SA of the two LD/BZ hollow cylinders. The LD/BZ release curve is very similar to PP1 (f_2_: 87.5). Here, 21% API is also released in 600 min. Due to the small outer SA of the PVA formulation in contact with the medium (24% of the SA), the separation of the layers could not proceed as quickly as desired, so that the increase in SA due to the separation of the hollow cylinders occurred late and thus did not lead to a faster API dissolution. In addition, it was observed that during printing of the PVA layer, EVA residues were still present in the print head, which were rinsed out despite the intermediate cleaning step and thus contaminated the PVA layer with EVA. The total density of the PP2 is 1.1 g/cm^3^. Despite the large shape, the dosage form floats on the medium, again most likely because of air entrapped in the structure (Figure 9). If the PDM-PVA layer between the LD/BZ-EVA-hollow cylinders dissolves, both parts (hollow cylinders with LD/BZ-EVA) float on the surface of the medium.

**Figure 10:**
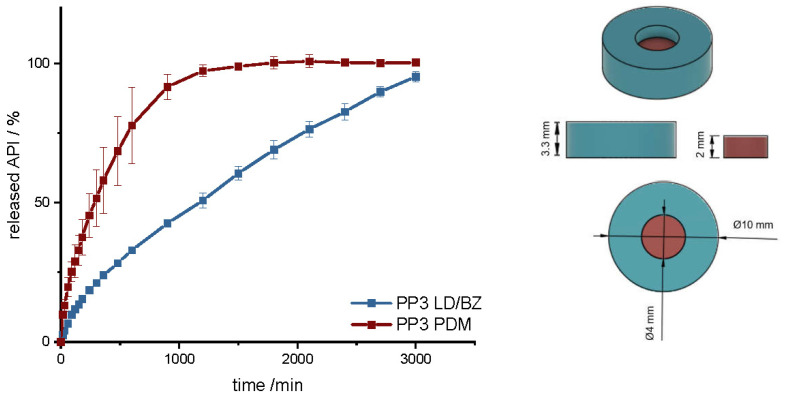
(**Left**): release profile of PP3; modified basket apparatus, 1000 mL 0.1 N HCl, 50 rpm, 37.0 ± 0.5 °C, x ± s; *n* = 3. (**Right**): Image of PP3: red: PDM-PVA, blue: LD/BZ-EVA.

In another polypill design (PP3), the PDM dose was changed. The total SA/V ratio was kept similar to PP1 and PP2. PP3 design was a hollow cylinder, this time with a small cylinder as inlay printed with the PDM-PVA filament (Figure 10). PDM-PVA was low-dosed with 1.5 mg and LD/BZ-EVA had a content of 50/12.5 mg (Table 9). 

To represent the complete release profile, the time of the dissolution test was extended to 3000 min. In this design, PDM was released very slowly. Despite a comparable SA/V ratio to PP1 and PP2, only 75% PDM was released within 600 min. The SA in contact with the medium was limited to 50%, so the SA in the complete design was reduced by the hollow cylinder from the EVA formulation. In addition, a filament change had to be performed for every single layer in this geometry, which again resulted in carryover of EVA into the PVA layers. For the LD/BZ-EVA formulation, a constant drug release after the burst could be realized with this design. With a release rate of 0.03% API/min, the release profile is comparable to PP1 and PP2, which was desired with the choice of SA/V ratio (f_2_: 60.1). The total density of the PP3 is 1.1 g/cm^3^. It also floats on the surface of the medium and maintains this property over the time of release.

With these geometries, it is possible to achieve a prolonged gastro-retentive API release for various dosages, which allows a larger time interval for drug absorption. In addition, due to the different geometric forms but comparable SA/V ratios, it is possible to vary the dosages from 50/12.5 mg–200/50 mg LD/BZ but keep the release profile similar (f_2_ > 50). However, the release profile of the LD/BZ combination is very slow (75% LD/BZ in 2100 min). For patients who need to respond more specifically to LD/BZ spikes, a 24 h ingestion-interval is not an option. In addition, the selected tablet sizes are not advantageous for patients with swallowing difficulties. Therefore, the possibility of printing mini tablets was also investigated in this study. 

### 3.5. Design and Dissolution of Polypill Mini Tablet Variations

With mini tablets, the dose can be finely adjusted by the patient himself by the selected number of mini tablets. Since the diameter is ≤5 mm, these dosage forms are easy to swallow [99,100].

**Figure 11:**
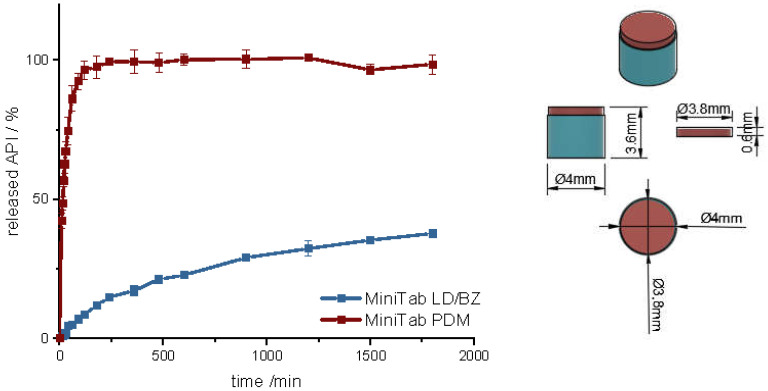
(**Left**): release profile of polypill design MiniTab; modified basket apparatus, 1000 mL 0.1 N HCl, 50 rpm, 37.0 ± 0.5 °C, x ± s; *n* = 3. (**Right**): Image of MiniTab: red: PDM-PVA, blue: LD/BZ-EVA.

First, a mini tablet (MiniTab) was printed with the dimensions of 4 mm in diameter and 3.6 mm in height (Figure 11 and Figure S1, Supplementary Material). The dose of LD/BZ was reduced to 15/3.75 mg per mini tablet, so the patient can adjust the desired LD/BZ dose in 15/3.75 mg steps by the number of tablets (Table 10). The dose of PDM was set to 0.375 mg, which represents the smallest dose in market preparations for SR. Therefore, the therapy can be adapted in small discreet steps.

The release of PDM is fast (100% PDM in 60 min). The small cylinder can be well covered by the medium, so that the API is quickly released from the matrix and the PDM-PVA cylinder can be well dissolved. The release of LD/BZ is again comparable to PP1-PP3 (f_2_: 80.3). 23% API was released in 600 min, and the release rate is 0.02% API/ min. With the MiniTab design, it would therefore be possible to reproduce the same release rate as with PP1-PP3, but the dose can be individually adjusted in small steps. In addition, PDM is released much faster with this form, so that any OFF phases of the patient can be treated quickly. Due to the low density (1.1 g/cm^3^), as well as the low volume likely in combination with entrapped air, this dosage form also floats on the surface of the medium and can thus be used as a gastro-retentive dosage form.

**Figure 12:**
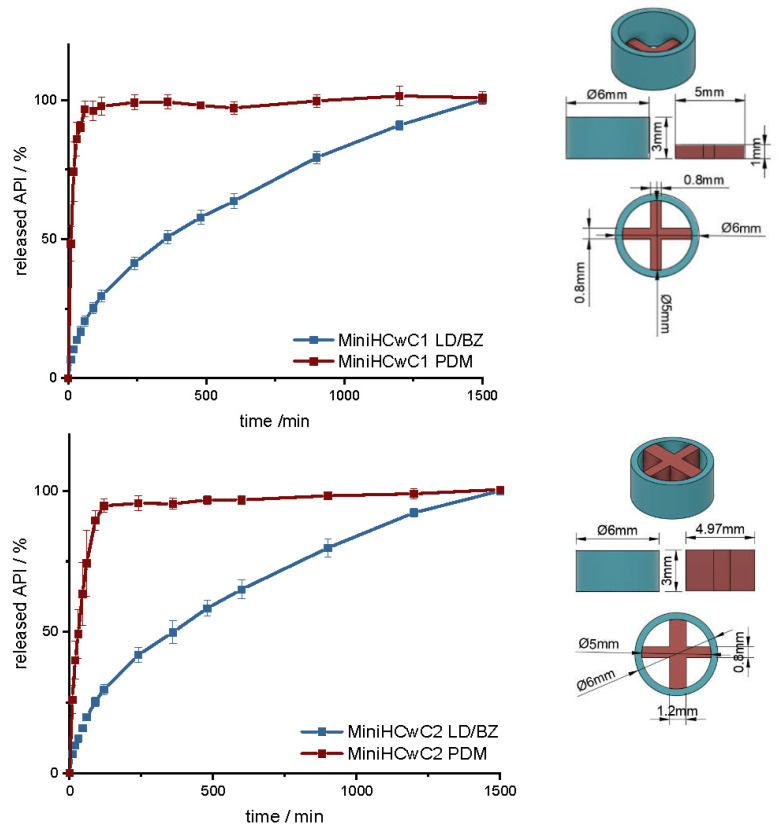
(**Left**): release profile of MiniHCwC 1 (**top**) and 2 (**bottom**); modified basket apparatus, 1000 mL 0.1 N HCl, 50 rpm, 37.0 ± 0.5 °C, x ± s; *n* = 3. (**Right**): Image of MiniHCwC1 + 2: red: PDM-PVA, blue: LD/BZ-EVA.

To increase the release rate of the LD/BZ combination, a SA/V ratio of 4.7 mm^−1^ was targeted with the next design. Therefore, a mini-hollow cylinder (MiniHC) with a dose of 10 mg LD and the appropriate SA/V ratio was printed. The interior was filled with a cross of PDM-PVA (Figure 12, Figures S2 and S3). This design allows for maximum circulation of the medium around both formulations. In addition, the dose of PDM can be varied by the height of the cross, or with a different design, which can be inserted into the hollow cylinder. The variation in height was tested with two different PDM-doses (Table 11). Figure 12 shows the release of MiniHC with cross with 0.4 mg PDM (MiniHCwC1, Top) and bottom shows the release of MiniHCwC2 with 1.5 mg PDM. 

**Figure 13:**
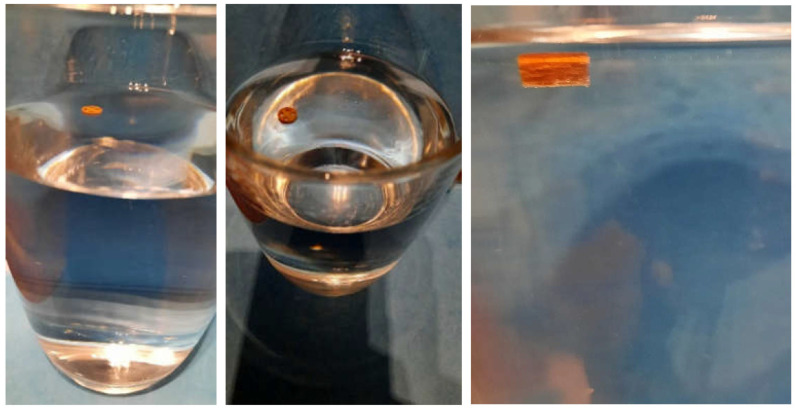
Floating properties of MiniHCwC in 300 mL 0.1 N HCl, 37 ± 0.5 °C.

The LD/BZ release shows the same dissolution profile in both MiniHC versions. First, a burst is seen; then, the API is released continuously over time at a rate of 0.07% API/ min. Due to the low wall thickness of the HC (1 mm) and the resulting short diffusion pathways for the APIs, the release profile remains constant over a long time and the release rate hardly decreases towards the end. With a released API fraction of 75% LD/BZ in 750 min, this release profile corresponds to the initially desired course. The PDM release is faster and differs in both variations. This was expected due to the various SA/V ratios. The printed cross with 0.4 mg PDM (PDM1, Table 11) has almost twice the SA/V ratio than the cross with 1.5 mg PDM (PDM2). Thus, the MDT is half as big, and the drug is released faster. This design makes it possible to insert various designs of other filaments, various SA/V ratios, and APIs, and to combine different release profiles. The inserted geometries can also be printed and inserted individually, independently of the outer hollow cylinder, so that there is no cross-contamination or mixing of the filaments. The floating property of the formulation allows prolongation of the GRT, a continuous release of the API and thus a saturation of the amino acid transporters in the upper small intestine section with LD (Figure 13). The small diameter and height, as well as the flexibility of the structure facilitate the swallowing of the 3D printed form for the Parkinson’s patients. This allows the therapy to be individually adapted to the patient.

## 4. Conclusions

In this study, the first printed oral dosage form with PDM/LD/BZ was developed. VCM was used as another new technology that is very useful to study the release properties of polymers without the influence of SA/V ratio. HME was used to prepare a fixed-combination of two drugs, and the FDM 3D printing process allowed the filament with the fixed-combination to be combined with another drug-loaded filament in variable dosages. In addition, the FDM 3D printing process enables variation of the SA/V ratio through the variety of possible geometries, as well as the incorporation of different layers and pores, all of which have an impact on the drug release process. Thus, not only the dose but also the onset and duration of the effect can be influenced. This approach makes it possible to address the individual needs of Parkinson’s patients, titrating the dose and increasing or decreasing it in small steps as needed. In this study, it was possible to increase the LD/BZ dose from 15–180 mg LD (3.75–107 mg BZ) and achieve a similar release profile (f_2_ > 50). In addition, mini tablets and mini hollow cylinders were printed, which might be easier for Parkinson’s patients to swallow and can be varied in number for ingestion so that the dose can be adjusted to the situation and the daily dose, to respond to ON-OFF-phenomena. Furthermore, the formulation has a low density, resulting in a floating property, which was used to prolong GRT. For drugs that are absorbed in the upper part of the small intestine, this increases the time of API absorption, and thus the medicine intake interval is increased. This improves patient adherence to their therapy.

The choice of polymer resulted in a very slow release; further studies may test whether the results can be achieved with other polymers. In addition, the polypill was prepared only with well water-soluble APIs. It would also be interesting to see how such a combination behaves with APIs of different BCS classes.

## Figures and Tables

**Table 1:** SR-polymers used for VCM-formulation development (MW, molecular weight; MFI, melt flow index).

	Polyvinyl Alcohol (PVA)	Hydroxypropyl Cellulose (HPC H)	Hydroxypropyl Cellulose (HPC SSL)	Ethylene Vinyl Acetate (EVA) (72:28)	Hydroxypropyl Methylcellulose Acetate Succinate (HPMC-AS)
**Manufacturer**	Parteck MXP^®^, 87–89% hydrolysis grade, MW: approx. 32,000 Da, Merck, Darmstadt, Germany	MW: 1,000,000 Da, Nisso Chemical Europe, Düsseldorf, Germany	MW: 40,000 Da, Nisso Chemical Europe, Düsseldorf, Germany	Escorene UL 02528^®^, MFI: 25 g/10 min, TER Chemicals, Hamburg, Germany	Aquasolve^®^, MW: 75,100 Da, Ashland, Wilmington, DE, USA

**Table 2:** Formulations used for the polypill.

**Filament 1**			
**API and Excipients**	**%**	**Function**	**Manufacturer/Source**
Pramipexole 2 HCl * H_2_O (PDM)	5.0	API	99.5%, Chr. Olesen, Gentofte, Denmark
Mannitol	10.0	plasticizer	Parteck M^®^, Merck, Darmstadt, Germany
Polyvinyl alcohol (PVA)	84.0	polymer	Parteck MXP^®^, Merck, Darmstadt, Germany
Fumed silica	1.0	glidant	Aerosil^®^ 200 VV Pharma, Evonik, Essen, Germany
**Filament 2**			
**APIs and Excipients**	**%**	**Function**	**Manufacturer/Source**
Levodopa (LD)	40.0	API	99.6%, Zhejiang Wild Wind Pharmaceutical, Dongyang, Zhejiang Prov., China
Benserazide (BZ)	10.0	API	99.8%, BioPharma Synergies, Barcelona, Spain
Vinylpyrrolidone-vinyl acetate copolymer 60:40 (PVP-VA)	15.0	polymer	Kollidon VA 64^®^, MW: 40,000 Da, BASF, Ludwigshafen, Germany
Ethylene-vinyl acetate copolymer 82:18 (EVA)	34.5	polymer	Escorene^®^ FL01418, MFI: 14 g/10 min, TER Chemicals, Hamburg, Germany
Fumed silica	0.5	glidant	Aerosil^®^ 200 VV Pharma, Evonik, Essen, Germany

**Table 3:** VCM-Process settings for different SR-polymers.

	PVA	HPC H	HPC SSL	EVA (72:28)	HPMC-AS
**Heating temperature/°C**	210	170	170	120	210
**Heating time/min**	7	7	8	7	7
**Mass/mg (MV)**	307	310	308	311	310
**SA/V ratio/mm^−1^**	1.5	1.5	1.5	1.5	1.5

**Table 4:** Extrusion parameters with adjusted temperatures during extrusion and screw configuration of performed extrusions.

**Temperature Profile in Zone 2–10 [°C]**									
	2	3	4	5	6	7	8	9	10
**PDM-PVA filament**	20	20	100	180	180	180	180	195	195
**LD/BZ-EVA filament**	20	20	100	100	100	100	100	100	100
**Screw Configuration (Die-Gear)**									
**PVA/EVA filaments**	die–10 CE 1 L/D–KZ 1: 5 × 60°–3 × 30°–5 CE 1 L/D–KZ 2: 4 × 90°–5 × 60°–3 × 30°–16 CE 1 L/D–2 CE 3/2 L/D–1 L/D adapter–gear CE = conveying element, KZ = kneading zone								

**Table 5:** Formulation development of SR LD-EVA formulation.

	F1	F2	F3	F4
**LD/%**	40	10	10	10
**EVA (72:28)/%**	60	44.5	-	-
**EVA (82:18)/%**	-	-	25	39.5
**PVA/%**	-	44.5	64	-
**Mannitol/%**	-	-	-	10
**PVP-VA/%**	-	-	-	39.5
**Fumed Silica/%**	-	1	1	1

**Table 6:** Formulation development of LD/BZ fixed combination (FC) formulation.

	FC1	FC2	FC3	FC4
**LD/%**	40	40	40	40
**BZ/%**	10	10	10	10
**EVA (82:18)/%**	44.5	39.5	34.5	29.5
**PVP-VA/%**	5	10	15	20
**Fumed Silica/%**	0.5	0.5	0.5	0.5
**Density/g/cm^3^**	1.15	1.16	1.17	-

**Table 7:** Structure and release properties of PP1.

	LD/BZ	PDM
**SA/V total/mm^−1^**	1.2	
**SA/V/mm^−1^**	1.6	2.4
**mg API/mg**	50.0/12.5	3.5
**% API in 600 min**	22	100
**t_75%_/min**	n.d.	140
**MDT/min**	n.d.	97
**release rate/%/min**	0.03	0.33

**Table 8:** Structure and release properties of PP2.

	LD/BZ	PDM
**SA/V total/mm^−1^**	0.9	
**SA/V/mm^−1^**	1.3	2.6
**mg API/mg**	83/20.75	3.5
**SA/V/mm^−1^**	1.2	-
**mg API/mg**	97/24.25	-
**%API in 600 min**	21	100
**t_75%_/min**	n.d.	310
**MDT/min**	n.d.	187
**release rate/%/min**	0.02	0.20

**Table 9:** Structure and release properties of PP3.

	LD/BZ	PDM
**SA/V total/mm^−1^**	1.1	
**SA/V/mm^−1^**	1.3	2.0
**mg API/mg**	50.0 / 12.5	1.5
**% API in 600 min**	33	75
**t_75%_/min**	2100	600
**MDT/min**	1130	360
**release rate/%/min**	0.03	0.11

**Table 10:** Structure and release properties of MiniTab.

	LD/BZ	PDM
**SA/V total/mm^−1^**	2.1	
**SA/V/mm^−1^**	1.6	4.4
**mg API/mg**	15/3.75	0.375
**% API in 600 min**	22.7	100
**t_75%_/min**	n.d.	40
**MDT/min**	n.d.	26
**release rate/%/min**	0.02	0.80

**Table 11:** Structure and release properties of MiniHC with a cross.

	LD/BZ 1 + 2	PDM 1	PDM 2
**SA/V total/mm^−1^**	-	4.7 with LD/BZ	3.7 with LD/BZ
**SA/V/mm^−1^**	4.7	4.6	2.9
**mg API/mg**	10/2.5	0.4	1.5
**% API in 600 min**	65	100	100
**t_75%_/min**	750	20	60
**MDT/min**	363	14	28
**release rate/%/min**	0.07	1.88	0.95

## Supplementary Materials

The following supporting information ca be downloaded at: https://www.mdpi.com/article/10.3390/pharmaceutics14050931/s1, Figure S1: Dimensions of *MiniTab*: 4.93 mm in diameter, deviation of 0.07 mm to the CAD model; Figure S2: Dimensions of *MiniHCwC*: 6.03 mm in diameter, deviation of 0.03 mm to the CAD model; Figure S3: Images of *MiniHCwC1* (left) and *MiniHCwC2* (right).
