# Supplementary material for: 3D Printed Mini-Floating-Polypill for Parkinson’s Disease: Combination of Levodopa, Benserazide, and Pramipexole in Various Dosing for Personalized Therapy
Source: Pharmaceutics. 2022 Apr 25;14(5):931. doi: 10.3390/pharmaceutics14050931 (PMC9145509; doi:10.3390/pharmaceutics14050931)
Supplement: Supplementary file 1 [file pharmaceutics-14-00931-s001.zip › pharmaceutics-1675096-supplementary.pdf]

3D Printed Mini-Floating-Polypill for Parkinson's Disease: Combination of Levodopa, Benserazide, and Pramipexole in Various Dosing for Personalized Therapy

Hellen Windolf, Rebecca Chamberlain, Jörg Breitkreutz, Julian Quodbach

Supplemental Material

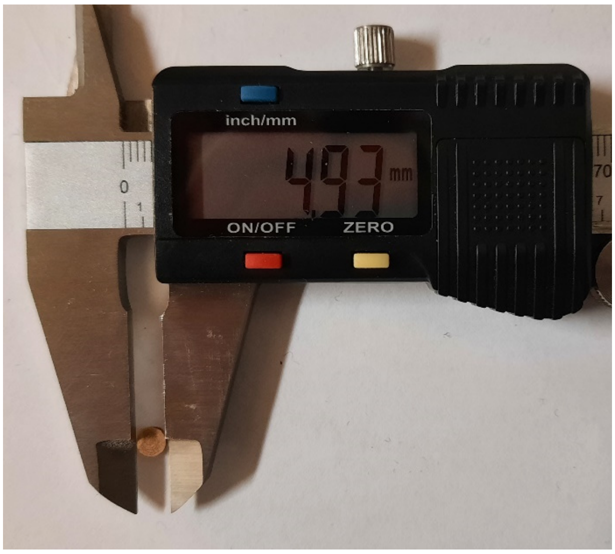

Figure S1: *Dimensions of MiniTab: 4.93 mm in diameter, deviation of 0.07 mm to the CAD model.*

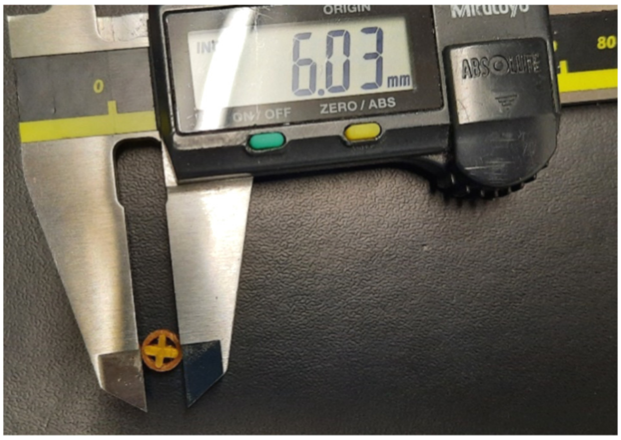

Figure S2: *Dimensions of MiniHCwC: 6.03 mm in diameter, deviation of 0.03 mm to the CAD model.*

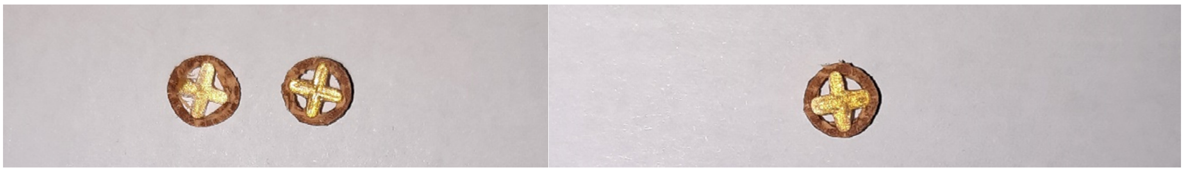

Figure S3: *Images of MiniHCwC1 (left) and MiniHCwC2 (right).*
